# Food safety in the Dominican Republic—The current situation and challenges in the public management system

**DOI:** 10.1002/fsn3.4311

**Published:** 2024-09-24

**Authors:** Silvia J. R. Vargas, Patricia Sipes, Silvia Tortosa la Osa, Paul Ebner

**Affiliations:** ^1^ Department of Animal Sciences Purdue University West Lafayette Indiana USA; ^2^ International Programs in Agriculture Purdue University West Lafayette Indiana USA; ^3^ Improving Economies for Stronger Communities, Trade Safe (TraSa) Project Santo Domingo Dominican Republic

**Keywords:** Dominican Republic, food safety

## Abstract

The Dominican Republic, like other economically developing countries, has begun to shift national focus away from food security to concentrate on improving food safety and nutrition. The government of the Dominican Republic has promoted and implemented measures aimed at eradicating poverty and malnutrition and reducing the diseases that are a direct consequence of nutritional deficiencies. However, between 2013 and 2018, the Dominican Republic reported approximately 23,000 cases of foodborne illness annually. Additionally, the number of annual cases of acute diarrhea has increased to approximately 80,000 cases per year with a mortality rate (per 100,000 inhabitants) between 5.98 and 6.94. While the etiological agents responsible for these cases are often not identified, foodborne pathogens including *Shigella*, *E*. *coli*, *Klebsiella pneumoniae*, *Salmonella*, and *Campylobacter* have been isolated from food exported from the Dominican Republic as well as locally consumed foods. This review describes the status of food safety in the Dominican Republic. The review focuses on what is known regarding the etiological agents involved in foodborne disease outbreaks in the country, the impact of foodborne disease, measures the Dominican Republic government has taken to ensure the safety of both exported foods and foods consumed locally, barriers to improving food safety, as well as current and emerging food safety challenges.

## Graphical abstract

With a fast‐growing economy, the Dominican Republic has focused efforts on improving the safety of foods eaten throughout the country. Here, we review the current state of food safety in the Dominican Republic with a focus on etiological agents responsible for outbreaks, different public and private sector initiatives to improve food safety, and suggestions as to how the country can most effectively advance food safety efforts.

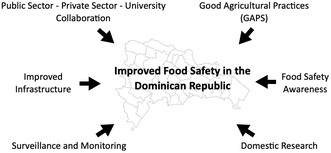

## 1 INTRODUCTION

Compared to neighboring countries in Latin America, the Dominican Republic (DR) has seen substantial economic growth in the past four decades, and in 2021, its economy grew at 4.7% (International Monetary Fund, 2021). Due to this economic growth, the DR, unlike many other countries in the region, is considered an upper‐middle income country according to the World Bank (World Bank, 2018), and is classified as having a high Human Development Index according to the United Nations (United Nations, 2022). Economic growth in the DR has been driven by a change in the productive structure of the country; whereas in recent decades the DR economy was driven primarily by agriculture and manufacturing, the DR economy has benefited from substantial growth in the service sector (Arrojo & Santos‐Paulino, 2013; Banco Central de la República Dominicana, 2022), as well as remittances and trade liberalization.

In 2016, the DR enacted Law 589‐16 on sovereignty and food and nutritional security, with the main objective of reducing hunger and poverty throughout the DR (Congreso de la República, 2016; Dirección General de Desarrollo Económico y Social, 2018). In turn, the general poverty rate was reduced from 49.55% in 2004 to 23.85% in 2021 (Ministerio de Economía Planificación y Desarrollo [MEPyD], 2021). While poverty rates have decreased, income inequality has increased. The impact of income inequality is felt more in rural areas considering the marginal reduction of the Gini coefficient of inequality between 2016 and 2021 (0.437–0.401) compared to urban areas (0.389–0.357); in absolute terms, the Gini coefficient continues at a value close to 0.40 in rural areas, while in urban areas it is 0.36 (MEPyD, 2021).

In terms of health security, however, mortality rates in the DR in children under 5 years of age are 3.4%, which is more than double that of Latin America and the Caribbean (1.6%; United Nations Inter‐agency Group for Child Mortality Estimation [UN IGME], 2021). Diarrheagenic diseases are the leading causes of early childhood mortalities. The lack of epidemiological data (e.g., etiological agents associated with these outbreaks/illnesses) makes it difficult to accurately ascertain which of these cases of diarrhea would be classified as foodborne illnesses. The lack of consensus on what constitutes a foodborne illness versus other types of gastroenteritis further complicates estimates. Nevertheless, conservative estimates suggest that the diarrheagenic diseases caused by consumption of contaminated food significantly affect health outcomes throughout the country (Organización Panamericana de la Salud / Organización Mundial de la Salud [OPS/OMS], 2022).

This review aims to provide an overall assessment of food safety in the DR. To achieve this goal, we focused on specific aspects of food safety including the etiological agents involved in foodborne disease outbreaks in the country, the impact of foodborne disease on different segments of the population, measures adopted by the DR government to ensure the safety of both exported and locally consumed foods, barriers to improving food safety, as well as current and emerging food safety challenges.

## 2 METHODOLOGY

To produce a narrative and integrative review of the status of food safety in the DR, we collected and reviewed related literature published in the last 10 years. The search was carried out between March 3 and April 29, 2022 using Web of Science, Scopus, Wiley Online Library, Embase, Google Scholar, and PubMed databases. Keywords and truncation symbols (*) were used in searches with the aim of increasing the search margin. Websites of organizations such as the Ministry of Public Health and Social Assistance of the DR, the US Centers for Disease Control and Prevention (CDC), the World Health Organization (WHO), the Pan American Health Organization (PAHO/WHO), the Food and Agriculture Organization of the United Nations were also consulted. In total, after removing duplicate records, 3785 records were obtained using the search terms “Dominican Republic”* AND food* AND safety* OR contamination OR poison* OR intoxication OR pathogen OR gastrointestinal disorder OR infection* OR hygiene* OR sanitation* OR “waterborne” OR diarrhea* OR vomiting* OR “Foodborne illnesses”* “Foodborne disease outbreaks” OR “Chemical products”* OR “chemical residues”.

### 2.1 Mechanism and selection criteria

**FIGURE 1:**
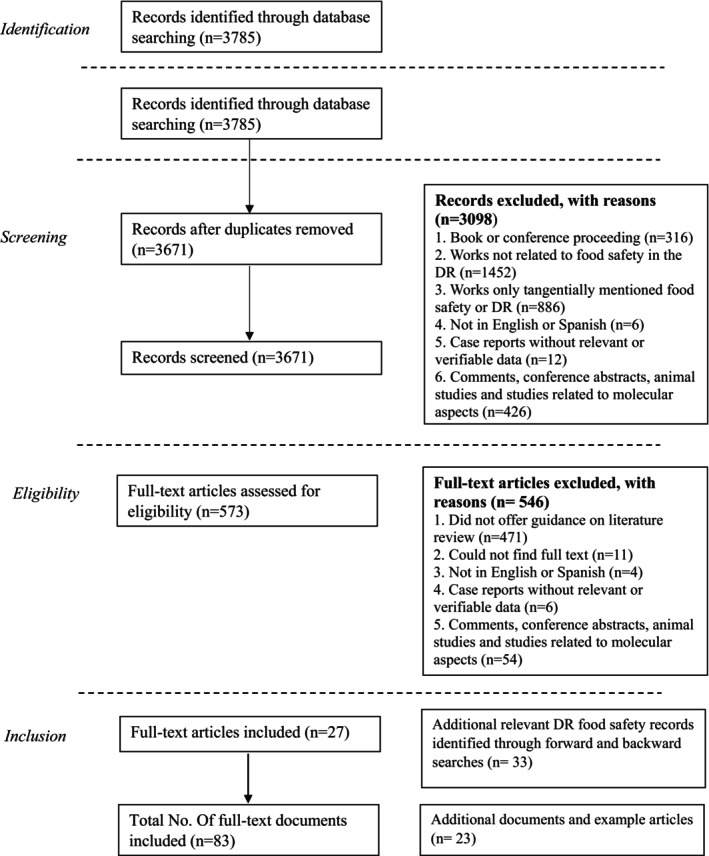
Flowchart for selecting articles and documents included in the review.

Initially, each article underwent a single‐author review. Inclusion and exclusion criteria were established to filter papers using the guidelines of Garcia et al., 2019. Specifically, the inclusion criteria (IC) used were:

- IC.1: Studies, reviews, and reports (governmental and nongovernmental) related to food safety in the DR;
- IC.2: Studies, reviews, and reports published online in the time frame after 2012 until 2022 (to contextualize food safety in the DR, some references prior to 2012 were used in the introduction of this review);
- IC.3: Studies, reviews, and reports published in Spanish and/or English;
- IC.4: The search terms were used to identify publications in Web of Science, Scopus, Wiley Online Library, Embase, Google Scholar, and PubMed databases, Websites of organizations such as the Ministry of Public Health and Social Assistance of the DR, the US Centers for Disease Control and Prevention (CDC), the World Health Organization (WHO), the Pan American Health Organization (PAHO/WHO), the Food and Agriculture Organization of the United Nations (FAO). The article selection process is described schematically in Figure 1.

After the exclusion process, 27 documents were obtained and each one was analyzed individually to extract the relevant data related to: prevalence of foodborne diseases, the involved etiological agents, government food safety measures, identified challenges and proposed solutions, relevant statistics, and changes in trends over time. If the analyzed document addressed one or more of these aspects, it was deemed as relevant. After the inclusion of additional relevant material and example material, there were a total of 83 documents included in the review.

Despite our efforts to include a broad spectrum of research, we acknowledge certain limitations in our approach. Specifically, our search predominantly focused on documents in Spanish and English, potentially introducing a linguistic bias to our findings. Additionally, our geographical scope was confined to the Dominican Republic, possibly limiting the generalizability of our results to other regions. However, this geographic restriction was intentional, aligning with the specific nature of our study on food safety in the Dominican Republic. While these constraints were purposeful to meet our primary objective, we recognize their potential impact on the overall representativeness of our review.

## 3 RESULTS

### 3.1 Food security an updated concept

**FIGURE 2:**
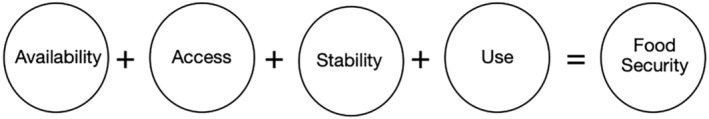
Evolution of the concept of food security (FAO, 1996).

Food safety is an integral part of food security. The definitions of food safety have evolved considerably over the past 30 years with both food safety concepts and food safety practices changing based on new knowledge at scientific levels and increasing demands at consumer levels (Figure 2). When first widely introduced in the 1970s, the concept of food safety, however, was not strongly tied to food availability and food security in general. In the mid‐1980s, however, food security efforts began focusing more on improving self‐sufficiency and access to food. Finally, at the 1996 World Food Summit, safety of food was also included as a major determinant of food security. According to the Food and Agriculture Organization (FAO), food security currently refers to individual's and group's physical, social, and economic access to sufficient, safe, and nutritious food that meets their dietary needs and food preferences for an active and healthy life (FAO, 1996), while food safety refers to the practices that are carried out during the production, handling, processing, and distribution of food to ensure that contaminants that can cause foodborne illness are not present. Therefore, food safety is transversal to food security and must be considered to truly achieve food security (FAO, 2022a).

The DR has been associated with the Food and Agriculture Organization of the United Nations (FAO) since the organization's inception in 1945 and the country became an FAO Member Nation in 1979. Since then, the DR has created numerous initiatives to improve the nutrition and food security of its citizens. The DR's 1994 constitution established that the state was the guarantor of food in its territory (Political Constitution of the Dominican Republic, 1994). However, in 2010, the country adopted Article 54 in which food safety was established as a right of its citizens (Political Constitution of the Dominican Republic, 2010). Although the country has made progress in the development of institutions, policies, and programs for food safety and security, the DR is still far from reaching the goals established in the UN Sustainable Development Agenda for 2030 (General Assembly, 2015). The comprehensive assessment of the real impact of the Dominican Republic's commitment to food security and safety requires specific data on the reduction of cases of diarrheal diseases in the population, and information on the etiological agents associated with acute diarrheal diseases, among other factors. Unfortunately, these data are not available in a digitized and virtual form from official organizations. Despite this limitation, a specific data point has been cited regarding the rate of change in mortality among children under 5 between 1990 and 2015 (−3.47 (−4.32 to −2.69)), as reported by Wang et al. (2016). Herrera et al. (2018) referenced these data to underscore the similarity in the incidence and mortality rates associated with diarrheal diseases in the Dominican Republic, emphasizing that this similarity could be due to better control of diarrheal diseases. However, this isolated data point is insufficient for a comprehensive assessment of the impact of the commitment to food safety and security in the country.

### 3.2 Food safety surveillance—current legislation in the Dominican Republic

In 2012 and 2016, in an interest to strengthen food and nutritional security, the government of the DR established Law 1‐12 National Development Strategy 2030 and Law 589‐16, respectively. These laws led to the establishment of the National System for Food and Nutrition Sovereignty and Security of the Dominican Republic (SSAN by its acronym in Spanish) in 2016 (Congreso de la República, 2016). These laws act as legal instruments to promote and implement measures aimed at eradicating poverty and malnutrition and reducing associated diseases, with priority given to the most vulnerable sectors and populations (Dirección General de Desarrollo Económico y Social, 2018).

Additionally, the DR established jurisdiction for food quality through different laws and decrees used in the Ministry of Public Health and Social Assistance (through the Department of Food and Beverage Surveillance) and the Ministry of Agriculture (through the Department of Agri‐Food Safety, Directorate of Animal Health and Department of Plant Health) (Decreto N^o^ 217‐91, 1991, 2001; Ley Del Sistema Dominicano Para La Calidad, No. 166‐12, 2012; Ley General de Salud, No. 42‐01, Art. 125, 2001; Ley Sobre Sanidad Vegetal, No. 4990, 1958). Together, these ministries or institutions implement chemical (e.g., pesticide residues, veterinary drugs, heavy metals) and microbiological (foodborne pathogens) surveillance and monitoring systems to varying degrees and efficacy on live animals (farm level), processed animal‐sourced foods, fruits and vegetables (farm, processing, and consumer levels), and agronomic crops (processing level). In some cases, the previously mentioned ministries or institutions have enforcement powers when a food matrix is found to be out of compliance with established standards. The results of such programs, however, are largely not publicly accessible (e.g., digitized, available via Internet, etc.) which makes evaluation of their efficacy in meeting their objectives difficult to measure (personal observation).

**FIGURE 3:**
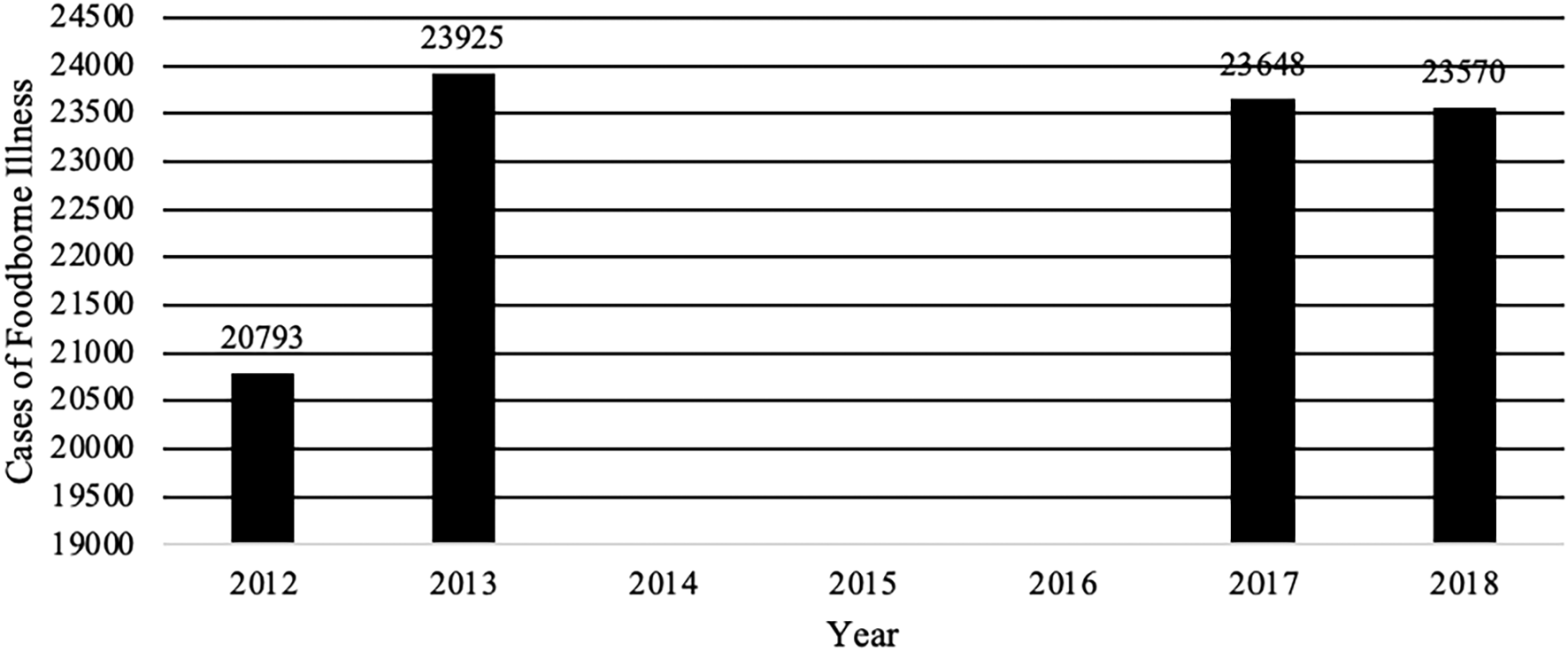
Cases of foodborne illness in Dominican Republic between 2012 and 2018 associated with food and water consumption. 2014–2016 No data reported. Data extracted from the epidemiological reports weeks of the Ministry of Public Health of the Dominican Republic, consulted 4/25/2022 https://digepi.gob.do/documentos/.

**FIGURE 4:**
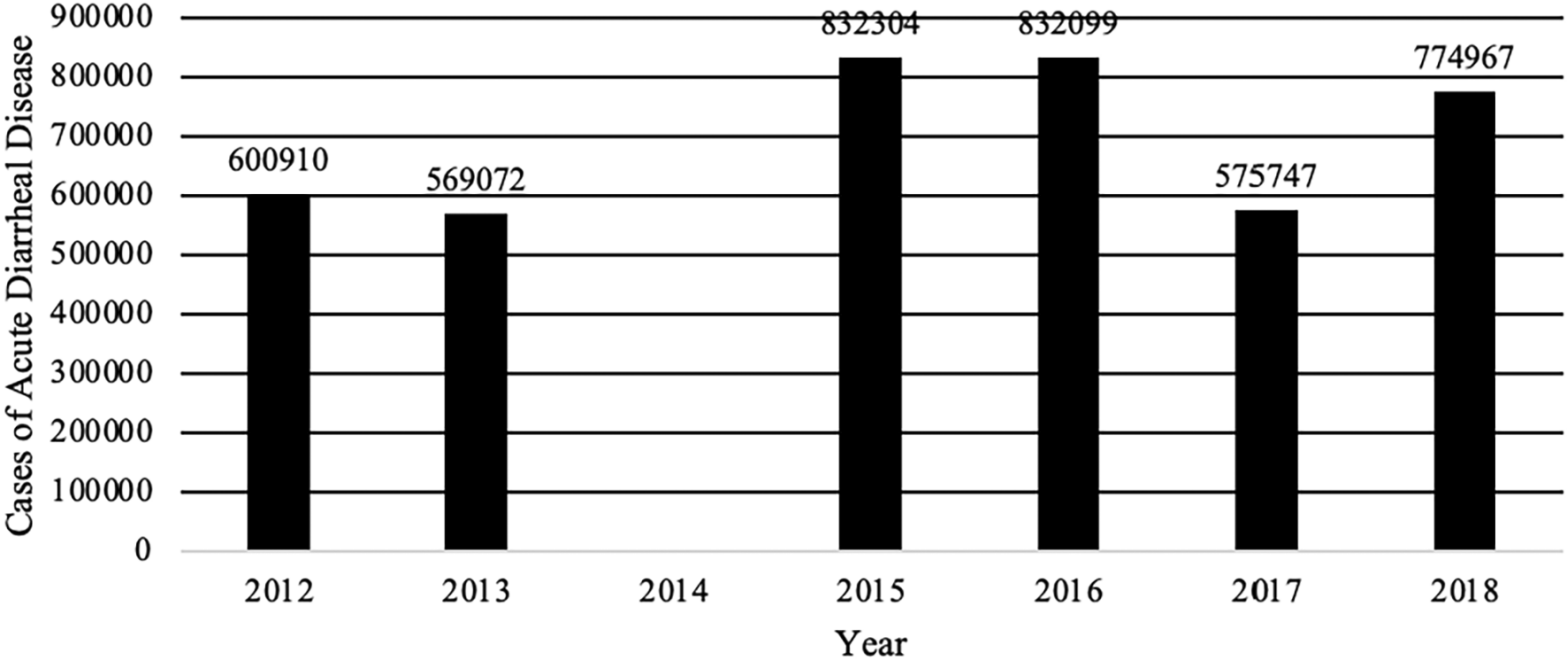
Cases of acute diarrheal disease in Dominican Republic 2012–2018 associated with food and water consumption. 2014 No data reported. Data extracted from the epidemiological reports weeks of the Ministry of Public Health of the Dominican Republic consulted 4/25/2022 https://digepi.gob.do/documentos/.

**FIGURE 5:**
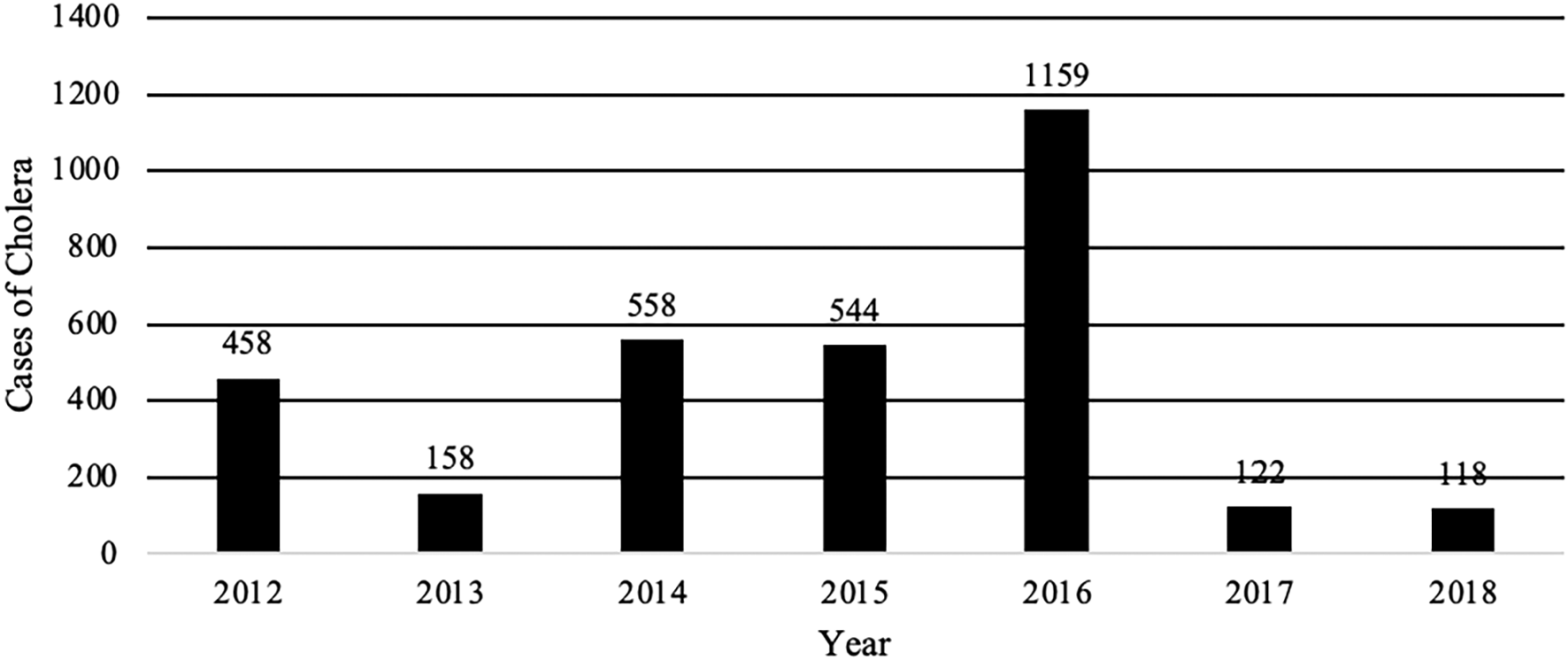
Cases of cholera in Dominican Republic 2012–2018 associated with food and water consumption. Data extracted from the epidemiological reports weeks of the Ministry of Public Health of the Dominican Republic, consulted 4/25/2022 https://digepi.gob.do/documentos/.

**FIGURE 6:**
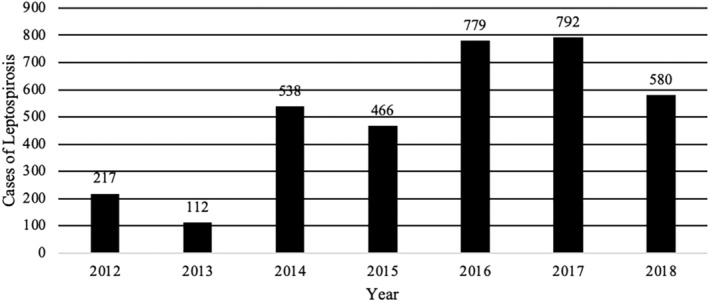
Cases of leptospirosis in Dominican Republic 2012–2018 associated with food and water consumption. Data extracted from the epidemiological reports weeks of the Ministry of Public Health of the Dominican Republic, consulted 4/25/2022 https://digepi.gob.do/documentos/.

Despite the existence of different food safety monitoring and surveillance programs, the DR continues to produce indicators that show high numbers of foodborne disease outbreaks and cases (Figures 3, 4, 5, 6). The data used for these figures are found in the Supporting Information (Table S1). From 2013 to 2018, the DR reported an average of 23,000 cases of foodborne illness annually. The number of annual cases of acute diarrhea, however, has grown to approximately 80,000 per year. In most cases of both foodborne illness and/or diarrhea, etiological agents were not identified. It is safe to assume, however, that many of the cases of acute diarrhea were associated with the consumption of contaminated food. Mortality rates (annual number of deaths per 100,000 people) associated with diarrheal diseases in the DR were 6.94 to 5.81 from 2013 to 2018, respectively (Dattani et al., 2023). For comparison, mortality rates associated with diarrheal diseases in the United States and the United Kingdom during the same period were 2.15 to 1.93 and 1.11 to 0.93, respectively (Dattani et al., 2023).

**FIGURE 7:**
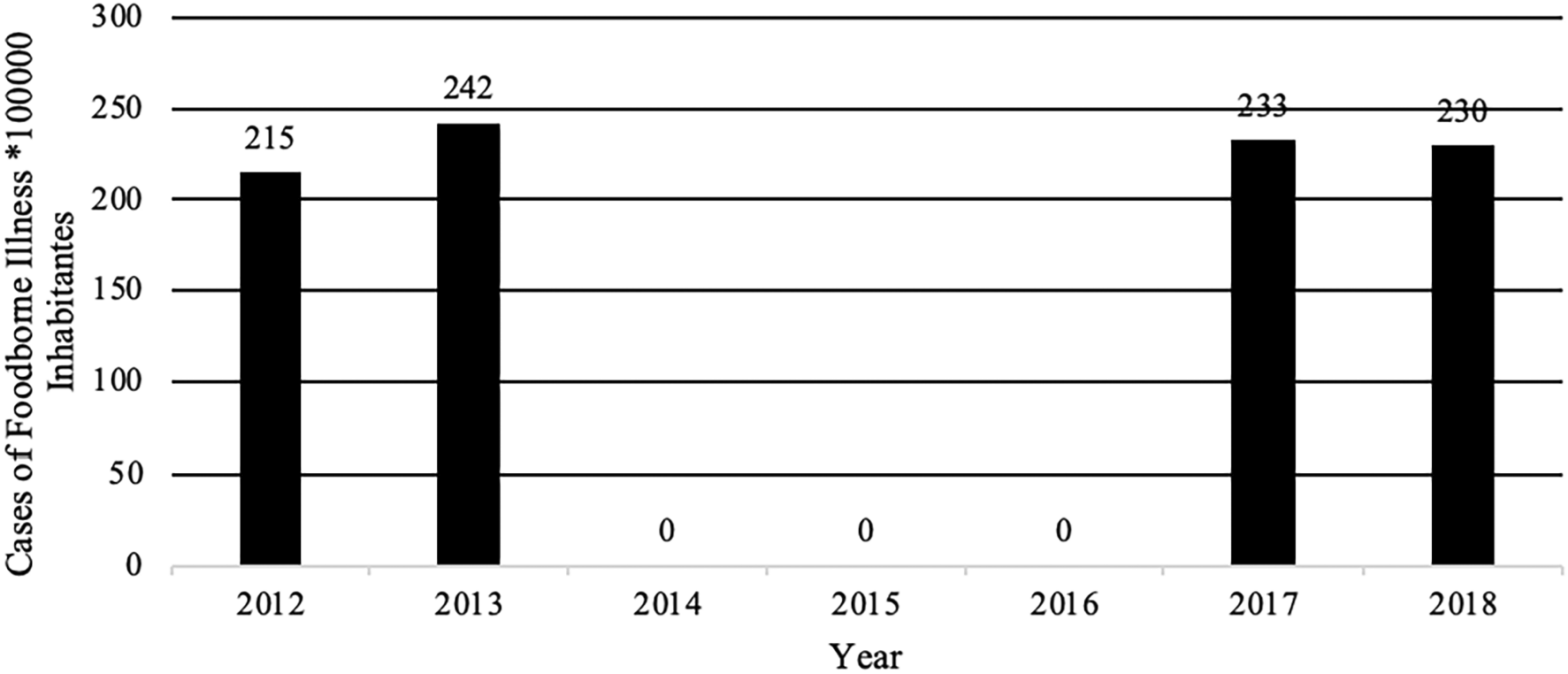
Prevalence of foodborne illness × 100,000 inhabitants in Dominican Republic 2012–2018. 2014–2016 No data reported. Data extracted from the epidemiological reports weeks of the Ministry of Public Health of the Dominican Republic, consulted 4/25/2022 https://digepi.gob.do/documentos/. Data on the number of inhabitants per year extracted from the National Statistics Office in the DR (ONE). 0 = no data.

**FIGURE 8:**
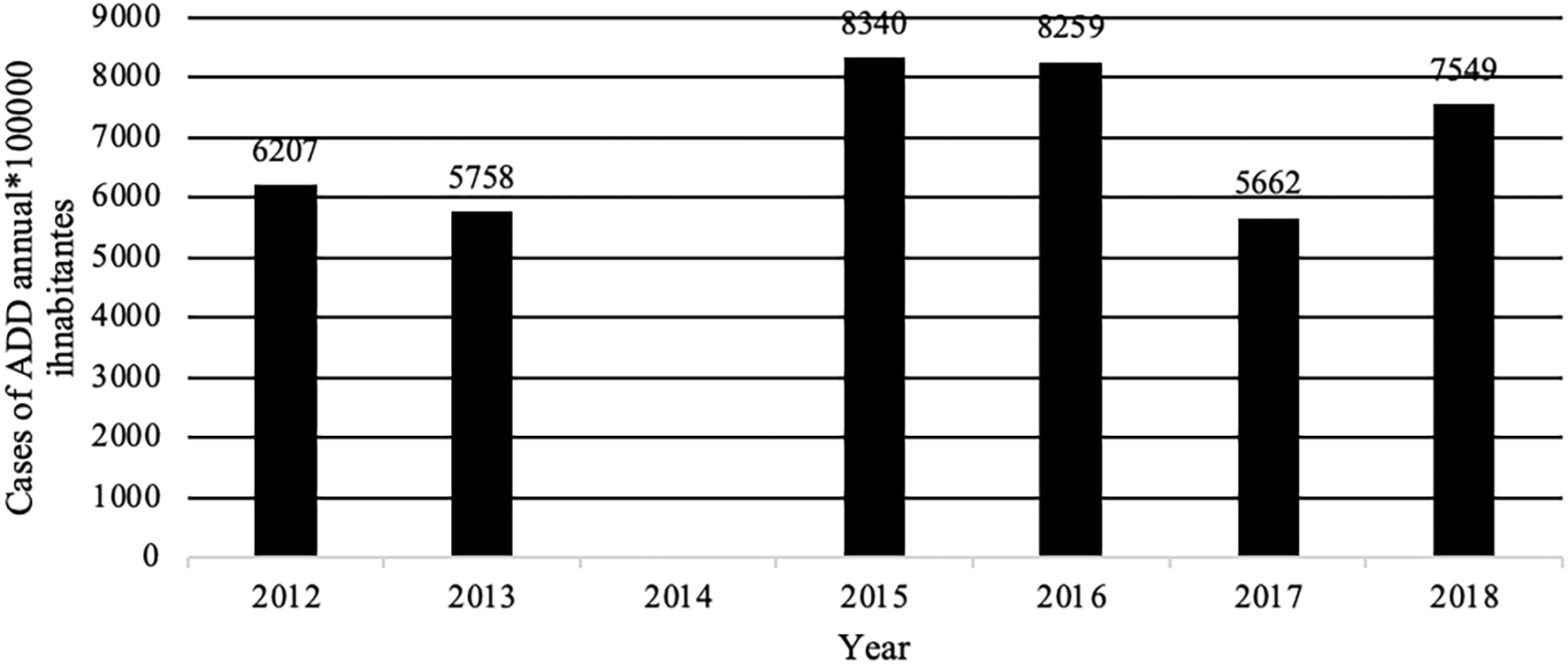
Prevalence of acute diarrheal disease × 100,000 inhabitants in Dominican Republic 2012–2018. 2014 No data reported. Data extracted from the epidemiological reports weeks of the Ministry of Public Health of the Dominican Republic consulted 4/25/2022 https://digepi.gob.do/documentos/. Data on the number of inhabitants per year extracted from the National Statistics Office in the DR (ONE); ADD, acute diarrheal disease.

More specifically, epidemiological indices (EI; number of cases observed during a study period compared to number of expected cases for the same study period) for foodborne illness cases between 2012 and 2018 never exceeded 1.25. EI values between 0.75 and 1.25 are generally acceptable and indicate that cases are within expected endemic levels (Alonso et al., 2007). However, it is important to note that in 2018, the EI in the 52 weekly reports for acute diarrheal disease in the DR ranged between 0.77 and 0.98 with 774,967 cases and the EI for foodborne diseases between 0.81 and 1.1 with 23,570 cases (Sistema Nacional de Vigilancia Epidemiológica, 2018). Additionally, while EIs were within the expected range, it is possible that, like most countries, the DR experiences large numbers of unreported cases of foodborne illness and/or acute diarrhea cases. In 2014, the Ministry of Public Health and Social Assistance did not report the specific number of cases of acute diarrheal diseases in its weekly bulletins and began to use, albeit inconsistently, the value of the weekly cumulative incidence of acute diarrheal disease per 10,000 or 100,000 inhabitants or the value of the epidemic index. This change in reporting made it difficult to consolidate annual data to show the real effect of breaches in food safety. To normalize the data presented, the number of annual cases per 100,000 inhabitants per year was calculated from the data extracted from the available data in weekly epidemiological reports of the Ministry of Public Health and Social Assistance of the DR (Figures 7 and 8). The data used for these figures are found in the Supporting Information (Table S2). Furthermore, between 2019 and 2023, the number of cases of foodborne diseases or waterborne diarrheal diseases specifically was not reported and the report was limited to indicating in its 52 annual reports that the IE was within what was expected range compared to the same period of the last 5 years. This data presentation format makes it difficult to know specifically the real impact that these diseases have on the population (Sistema Nacional de Vigilancia Epidemiológica, 2022). Taken together, however, these data indicate that despite the existence of control agencies and programs to improve food safety, the DR still experiences a comparatively high number of foodborne illnesses each year. In addition to observable health impacts, foodborne illness and/or acute diarrheal disease exact an economic burden. Among the most vulnerable populations alone, children under 5 years of age, costs associated with foodborne illnesses are estimated to exceed 46 million dollars a year to the DR (Prost & Martínez, 2019).

### 3.3 Condition of availability and sufficiency of food

As shown in FAO's 2021 report on the state of food security and nutrition in the world, income inequality increases the likelihood of food insecurity (FAO, FIDA, OMS, PMA, 2021). According to the DR Ministry of Economy, Planning and Development (MEPyD), the national poverty rate was 23.85% for 2021, which represents an increase of 0.46% compared to the same period in 2020. For comparison, the DR national poverty rate is more than double that of the United States (11.4% in 2020; Shrider et al., 2020). Extreme poverty in the DR in 2021 was estimated at 3.06%, which was down 0.44% points compared to 2020. Extreme poverty is defined by the World Bank as existing on less than $1.9 USD per day and corresponds to the poverty line in USD in power parity conversion factors (PPP) updated in 2011 (Ferreira et al., 2016). Individuals experiencing extreme poverty may also have very limited access to basic necessities such as food, clean drinking water, sanitation, shelter, and clothing. As in most nations, poverty in the DR is more prevalent and acute in rural areas (MEPyD, 2021). As such, rural households are more likely to face food safety and security problems.

Food security is often a function of a population's ability to purchase food. Gomez Campusano and García Tamez (2021) investigated food accessibility of Central American countries and the DR and reported that Dominicans require 1.32 times the minimum wage to purchase the total food basket (CBT [Spanish acronym]). As such, Dominicans spend an estimated 33% of their average monthly income on food. Comparatively, individuals in the United States utilize approximately 10% of disposable personal income for food, a value that has not changed since 2001 (Zeballos & Sinclair, 2022). Food costs as percentages of disposable income in the United Kingdom are similar to those in the United States (Department for Environment Food & Rural Affairs—UK, 2021).

The consequences of food insecurity in the DR go beyond malnutrition, which is seen in approximately 9% of the population. For example, 26.4% of women of reproductive age experience anemia, 11.3% of newborns are underweight, and 26.7% of adults are obese, as are 7.6% of children under the age of five (FAO et al., 2021).

Income levels and population shifts have also contributed to various nutritional outcomes. DR has made significant progress in reducing malnutrition from 1.8 million people in the 2000–2002 period to 0.9 million people between 2018 and 2020. Additionally, according to data from the National Statistics Office, the urban population of the DR grew 7.5% between 2012 and 2022 (Oficina Nacional de Estadística [ONE], 2015), and per capita income rose 8.5% between 2010 and 2021, mostly in urban residents (MEPyD, 2021). Urban growth has largely been the result of economic factors and shifts in major industries from agricultural production to services and tourism (Díaz, 2018). These changes have resulted in greater demands for food as well as a transformation in the pattern of consumption, moving from a diet based fundamentally on cereals, tubers, vegetables, and fruits, toward a diet with greater consumption of sugars, fats, and processed or ultra‐processed foods (del Rosario, 2021). As seen with other countries, the new consumption patterns favor diseases associated with overnutrition and obesity (FAO et al., 2021).

### 3.4 Relevance of food safety

Seeking to improve the food security of the most vulnerable populations, the DR has initiated numerous training programs for farmers, producers, and food processors in the country. Central to these efforts is the Inter‐American Institute for Cooperation on Agriculture which provides training on issues related to phytosanitary protection, food safety, and responsible production, while also implementing programs that seek to improve food and nutritional security (Inter‐American Institute for Cooperation on Agriculture [IICA], 2014). In its 2017 report, IICA reported its collaboration with the Commonwealth of Greater Santo Domingo (MGSD) and with the National Federation of Merchants and Entrepreneurs of the Dominican Republic (FENACERD) in the preparation of a proposal to improve food security and safety of food in the markets of greater Santo Domingo through a network of municipal governments, with the active participation of 25 businessmen and representatives of the municipalities. Additionally, this collaboration contributed to the reported improvement in the technical capacity of 56 professionals from the public and private sectors through participation in regional and international forums on agricultural health and food safety, competitiveness in agricultural chains, agriculture, and climate change, among others (Instituto Interamericano de cooperación para la Agricultura [IICA], 2017). Between 2018 and 2021, IICA has actively participated with government institutions and the private sector in the evaluation, improvement, and updating of agricultural health, and food safety in national services (, 2022; Instituto Interamericano de cooperación para la Agricultura [IICA], 2019). IICA currently has projects with a 2023 budget of more than 2.5 million dollars for the execution of technical cooperation projects that seek to strengthen institutions and rural business organizations as well as the improve capacities of the Ministry of Agriculture to support competitive, sustainable, and inclusive agricultural development and rural welfare in the DR.

### 3.5 Water

Water plays a key role in food safety, not only because it is a basic element in food, but also because of its role in the different stages of production and processing. Access to quality water in sufficient quantities facilitates safe food handling practices that prevent many foodborne infections (U.S. Food and Drug Administration, 2022). According to WHO (2019), at least 10% of the world population regularly consumes foods that have been produced or irrigated with water not suitable for human consumption. As such, fresh fruits and vegetables that come in contact with unsuitable irrigation water, especially those consumed raw, are generally considered high‐risk foods in terms of food safety (WHO, 2019). It follows that greater and better access to quality water sources, together with improved sanitation and sewage treatment, significantly reduces deaths from diarrheal diseases and substantially improves the quality of life of the population in developing regions (Ashbolt, 2004; Stauber et al., 2009).

According to the report of the government of the DR in 2021 on the progress of the agenda of the Sustainable Development Goals 2030, there is a significant gap in access to quality water between urban (93% access) and rural (76.8% access) populations. Confounding matters, five out of 10 households in rural and urban areas have intermittent access to quality water (between 1 and 6 days a week), a factor that is aggravated in rural areas by the poor quality of supplied water (World Bank, 2021a, 2021b). Similarly, access to sanitation services is profoundly unequal across income levels where 95.4% of households in the highest income brackets have basic sanitation such as drinking water supply, urban sanitation, sanitary sewage/storm drainage, and adequate solid waste management, while the same is true for only 71% of the poorest households (Comisión interinstitucional de alto nivel para el desarrollo sostenible, 2021).

Several groups have examined water quality in the DR. Baum et al. (2014) examined microbial profiles of water supply sources used in the Puerto Plata province of the DR. The authors found that 47% of improved drinking water sources were unsafe for human consumption based on *E*. *coli* concentrations. Thus, while 82% of the DR has access to improved drinking water sources, the low quality of water produced by these sources may contribute to the high incidence of diarrheal disease in the country either through direct consumption or through the use of such water in food production.

Using data from the 2007 Demographic and Health Survey of the DR (EDS‐DR), McLennan (2015) characterized the water consumption habits of 31,220 households. The authors reported that 59.6% of respondents used bottled water as their main source of water as compared to 24.7% of respondents who used tap water. Although the authors stated limitations in perceived water quality, the results suggest that many Dominicans perceive tap water sources as less fit for consumption compared to bottle water sources.

Tintle et al. (2021) conducted a randomized and controlled trial to determine whether the installation of membrane filters at drinking water sources in DR homes could reduce the incidence of diarrhea. The authors found that the use of membrane filters reduced the incidence of diarrhea from 25.6% to 9.76% with reductions seen across all age groups. However, McLennan (2016) also analyzed the water consumption habits of caregivers of children under 5 years of age between 2010 and 2013 in relation to the use of point‐of‐use (POU) strategies to improve drinking water during the cholera epidemic. The authors found that there were very few respondents utilizing practices to improve the quality of the drinking water provided to children.

Enhancing water quality in the Dominican Republic to ensure food safety necessitates specific measures. These measures would include strategic investments in water infrastructure, rigorous implementation of water testing and monitoring, adherence to water quality standards, and educational initiatives targeted at agricultural stakeholders to encourage the adoption of safe water practices.

### 3.6 Etiological agents related to foodborne disease outbreaks and their surveillance

The Ministry of Public Health and Social Assistance of the DR produces epidemiological reports on outbreaks of diseases transmitted by food and water. However, the reports do not usually contain information on the actual etiological agents responsible for the outbreaks. Outside of these reports, there is little additional information on the specific organisms responsible for foodborne illness outbreaks. The information gap has been noted by other groups as well (Palmieri et al., 2014). Likewise, there is little information on the types of foods associated with such outbreaks (Heredia & García, 2018).

Some investigations have linked agricultural products produced in the DR with etiological agents responsible for foodborne diseases. Zhi et al. (2021) showed that many of the outbreaks of shigellosis observed in Europe and the United States between 2003 and 2015 had a direct connection with the DR with about 50% of such cases linked with recent travel to the DR. Likewise, Gray et al. (2015) reported 11 cases of shigellosis in French tourists who reported recent travel to the DR, Haiti, or French Guiana. For their part, Zurfluh et al. (2015) isolated *Enterobacteriaceae* (*E*. *coli* and *K*. *pneumoniae*) in 33% of the 49 samples of fresh vegetables from the DR analyzed in their study. While a small sample, the study indicated that these pathogens are isolated from foods commonly consumed in the country.

The DR does have surveillance systems in place for key diseases, both nationally and regionally. The 2010 cholera outbreak in neighboring Haiti serves as an example of the functionality of these systems. The outbreak resulted in 21,432 cases and 363 deaths. Due to surveillance and control measures, however, the number of cases decreased to 13 in 2019 (Sistema Nacional de Vigilancia Epidemiológica, 2019). No cases of cholera were reported to the DR Ministry of Public Health and Social Assistance in 2020 and 2021 (Sistema Nacional de Vigilancia Epidemiológica, 2021). However, in 2022, 10 cases were reported and up to week 7 of 2023, 54 cases of cholera had already been reported (Sistema Nacional de Vigilancia Epidemiológica, 2022, 2023). Without directly relating the cases to the consumption of specific foods, Palmieri et al. (2014) showed that 127 of 128 fecal samples from children collected in the city of Verón in the DR were positive for one or more gastrointestinal parasites including *Ascaris lumbricoides*, *Giardia duodenalis*, *Entamoeba histolytica*, or *Enterobius vermicularis*. The authors did not identify possible sources of infection.

The WHO reported in 2017 that around 1.7 billion cases of childhood diarrheal diseases occur worldwide with 525,000 deaths annually in children under 5 years of age (WHO, 2017). In 2021, the DR experienced 34 deaths of children under 5 years of age per every 1000 births. For comparison, in the United States, this value is just six deaths for every 1000 births. The United Nations Inter‐institutional Group for the Estimation of Infant Mortality (UN IGME, 2021) suggests that the management of diarrheal diseases in children under 5 years of age should include the provision of quality drinking water and the improvement of basic sanitation and hygiene, which are basic food safety measures.

### 3.7 Food safety and use of chemicals

In the same way that microbial pathogens are a risk to food safety, the inappropriate or overuse of chemical products (e.g., pesticides, herbicides, antimicrobials, etc.) at different stages of food production also represents significant food safety risks (Smith, 1997; Wu et al., 2017). Silfrany et al. (2013) detected quinolone residues, a class of antibiotics used in both human and veterinary medicine, at higher than allowable limits in 50% of the 135 DR chicken samples analyzed. In 2015, Moscoso et al. (2015) also reported the presence of quinolones in eggs produced in 48 farms located throughout the DR. It is important to note that quinolones are not approved for use in egg‐producing poultry. These results indicate that greater control over the use of veterinary drugs is needed to ensure consumers are not exposed to these drugs or their active metabolites through the consumption of food animal products (U.S. Food and Drug Administration, 2016).

Similarly, exposure to pesticides or their residues through food consumption is another concern. Murray and Hoppin (1992) described how the lack of control over and the inappropriate or overuse of pesticides made these practices a potential danger for farmers, agricultural workers, exporters, and consumers. Hutter, Kundi, et al. (2018) compared the self‐reported health symptoms of 38 pesticide‐using farm workers in the Jarabacoa municipality of the DR with 33 organic farm workers from the same region, noting a higher incidence of acute symptoms of neurotoxic or parasympathetic effects such as excessive salivation, dizziness, irregular heartbeats and stomach pain in pesticide applicators. Hutter, Khan, et al. (2018) also reported cytological signs of genotoxicity in the same group of workers (38 pesticide applicators in the Jarabacoa region of the DR) using proportions of micronucleated cells (3.1; 95% confidence interval: 1.3–7.4) or karyolysis: 1.3 (1.1–1.5) which are markers of oral toxicity. Moshammer et al. (2020) reported possible endocrine effects affecting male fertility in workers who reported their first pesticide exposure before the age of 20. Although the authors considered other possible factors that could affect this result, the genotoxicity data are strong indicators of possible permanent effects.

### 3.8 Tourism and food safety in Dominican Republic

Foodservice and tourism account for approximately 26% of the DR gross domestic product (OECD/UNCTAD/ECLAC, 2020). Other sectors heavily rely on food service and tourism as well; 25% of the agricultural production in the DR is utilized in food service and tourism (Meyer, 2020). Several studies, however, have reported on foodborne illness cases and outbreaks associated with travel to the Dominican Republic.

Lόpez‐Vélez et al. (2022) reviewed characteristics of cases of bacterial diarrhea in travelers to Latin America and the Carribean between 2010 and 2020. The authors reported on the pathogenic organisms found in feces of patients who had traveled to the region, with *E*. *coli* being the most frequently isolated pathogen (0%–54% enteroaggregative *E*. *coli*: 0%–54%; enterotoxigenic *Escherichia coli*: 0%–60%; 0%–29%; enteropathogenic *E*. *coli*: 0%–29%); *Shigella*: 0%–27%; *Salmonella*: 0%–6%; and *Campylobacter*: (0%–13%). Additionally, while not usually considered a foodborne pathogen, *V*. *cholerae* was isolated from the feces of between 0% and 1.3% of patients.

Another review by Díaz et al. (2022), which included 22 studies reported between 1992 and 2017 of foodborne illness outbreak in the hotel sector of the DR, identified *Salmonella enterica* serotypes Enteritidis, Typhimurium, Newport and Javiana, *Campylobacter*, *V. cholerae*, and *Shigella* as among the most prevalent etiological agents (40%) overall in foodborne illness. The authors also identified etiological agents in foodbborne illness outbreaks associated with the consumption of fish with ciguatera (32%), *Toxoplasma gondii*, *Cyclospora cayetanensis*, and *Entamoeba histolytica* (14%), and Norovirus (14%) most frequently isolated. Johnson et al. (2011) used demographic and clinical information from the US Centers for Disease Control and Prevention (CDC) Foodborne Disease Active Surveillance Network (FoodNet) collected between 2004 and 2008 to analyze the relationship between cases of salmonellosis in people who returned to the United States from the DR. The authors reported that 2.5% of salmonellosis cases corresponded to people who had manifested symptoms of the disease up to 7 days after having visited the DR as their only destination.

Kendall et al. (2012) also used FoodNet data to analyze travel‐associated enteric infections between 2004 and 2009 and found that 3.2% of cases were in travelers returning from the DR, with *Campylobacter*, nontyphoidal *Salmonella*, and *Shigella* most frequently identified as etiological agents. While the exact number of cases per pathogen could not be calculated, the authors estimated that nontyphoidal *Salmonella* was responsible for approximately 60% of travel‐associated enteric infections in the Caribbean region.

Webb et al. (2022) using reports from state laboratories in the United States, reported 14 cases of traveler's diarrhea in individuals returning from the DR, caused by *Salmonella* carrying *mcr‐1* genes, which confer multi‐antibiotic resistance. The authors reported that patients developed symptoms of foodborne illness between 0 and 5 months after their return from the DR.

The Dominican Republic has undertaken technical assistance initiatives to enable producers to deliver safe food to the tourism sector and to promote sustainable tourism in an effort to uphold food safety requirements in facilities frequented by visitors (FAO, 2022b). The National Traceability System was implemented nationwide by the Ministry of Agriculture (Ministerio de Agricultura de la República Dominicana, 2013) as an extra precaution to guarantee the hygienic safety of meats. In the meantime, monitoring, directing, and guaranteeing adherence to regulations that safeguard consumers' health, especially those in the travel and tourism industry, fall within the purview of the Department of Food Safety (DIA) (Departamento de Inocuidad Agroalimentaria, s.f.) and the National Institute for Consumer Rights Protection (Pro Consumidor; Ley General de Protección de los Derechos al Consumidor o Usuario, No. 358‐05, 2005). This is achieved by regular audits, awareness campaigns, and food handler training programs.

### 3.9 COVID‐19 and its impact on food safety in the DR

Although the SARS‐CoV‐2 virus is not considered a foodborne pathogen, the measures adopted to combat COVID‐19 are often closely linked to safe production and handling of food (OECD, 2021). Additionally, the COVID‐19 pandemic had a direct impact on the economic and social condition of people, especially in developing and emerging economies, including the DR (MEPyD, 2021). First, food accessibility was affected by the economic crisis and the impact was greater on the poorest women. In the first year of the pandemic, 7.5% of Dominican women lost their jobs compared to 4.9% of men (Ministerio de Economía Planificación y Desarrollo [MEPyD], 2020). This gender difference is large when compared with unemployment rates in the United States between 2019 and 2020 that ranged between 3.6% and 6.9% for men and between 3.6% and 6.6% for women (Smith et al., 2021).

The monetary poverty rate, defined by the UN as the percentage of individuals or households whose income falls below a certain threshold level considered as the poverty line (UN, n.d.), increased from 21.0% to 23.4% in the DR in 2020 despite programs created by the government to mitigate the impact of falling incomes associated with the COVID‐19 pandemic (MEPyD, 2020). During the same period, the United States monetary poverty rate ranged from 10.5% to 11.4% (Shrider et al., 2020). Additionally, as in many countries, the DR has a significant informal food market. In many communities, most fresh foods are purchased through the informal market which may have little to no oversight or regulation. Such wet markets can provide an enabling environment for current and emerging zoonotic diseases. As such, informal markets require control and surveillance to both prevent foodborne diseases as well as events like COVID‐19 from repeating themselves with the same ease. However, the creation of food safety standards must consider local contexts and have a solid implementation infrastructure so that they can be successfully applied and maintained over time, especially in the poorest sectors where local markets provide access to affordable food for many communities (OECD, 2021).

### 3.10 Perception of food safety in agriculture in the Dominican Republic

**FIGURE 9:**
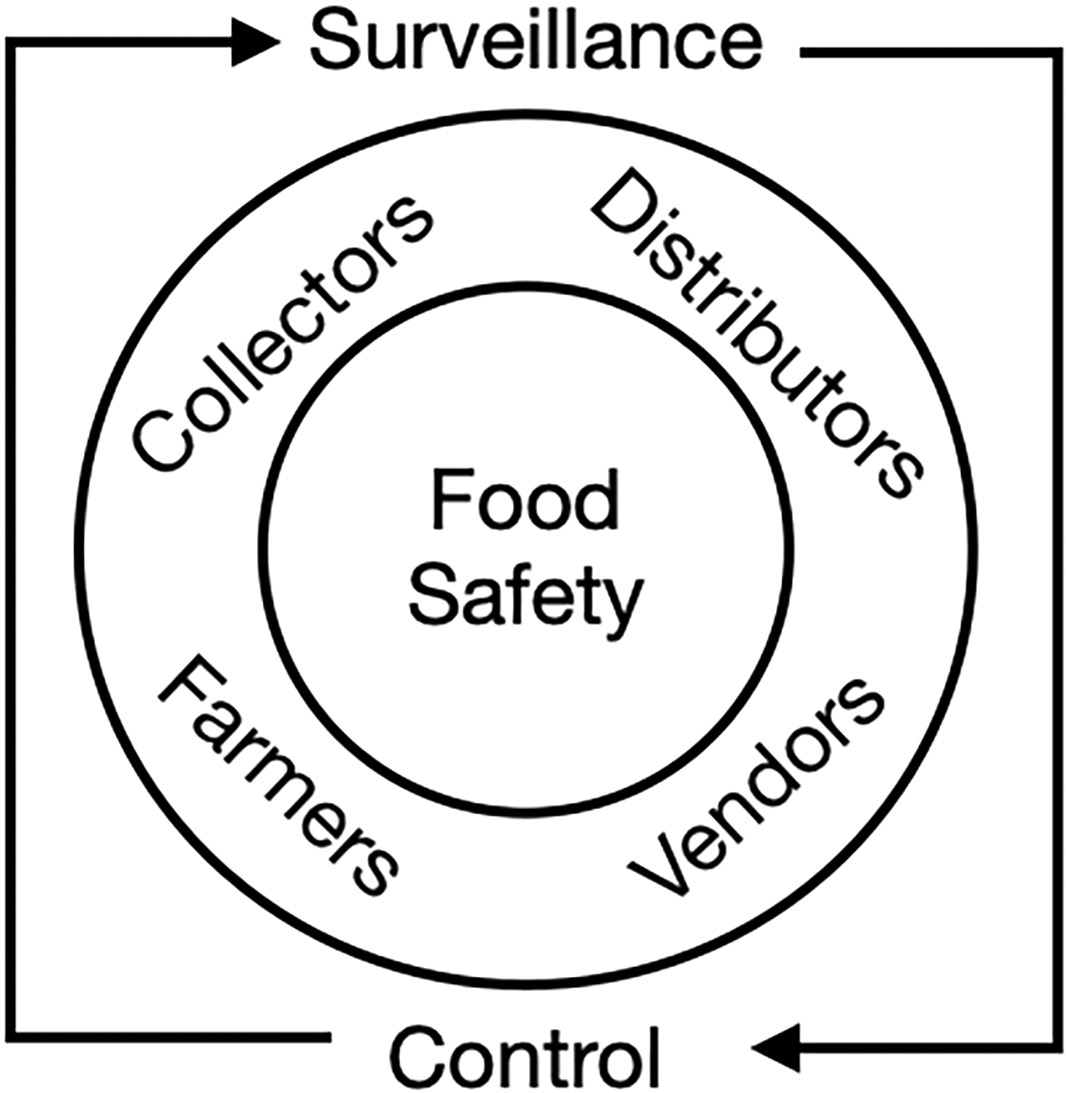
Orientation of the possible integrated management of surveillance programs that should include all entities involved in the agricultural production, distribution, and marketing systems to improve food safety in the Dominican Republic.

Although food safety has been addressed by different government agencies for several decades in the DR, the appearance of outbreaks of diseases transmitted by food and water in recent years (Table 1) demonstrates the need to establish more effective monitoring and control systems in the various stages of food production. Such systems should include all actors across production chains including farmers, processors, distributors, sellers, and, ultimately, consumers (Figure 9).

**TABLE 1:** Cases of diarrheal diseases associated with food and water consumption in the Dominican Republic between 2012 and 2018^a^.

Food and waterborne illnesses	2012	2013	2014	2015	2016	2017	2018
Acute diarrheal disease	600,910	569,072	–	832,304^b^	832,099^c^	575,747	774,967
Foodborne illness	20,793	23,925	–	–	–	23,648	23,570
Cholera	458	158	558	544	1159	122	118
Leptospirosis	217	112	538	466	779	792	580

*Note*: –, No data.

^a^ Data extracted from the epidemiological reports weeks of the Ministry of Public Health of the Dominican Republic, consulted 4/25/2022 https://digepi.gob.do/documentos/.

^b^ Value reported only in the weekly report N°52.

^c^ Sum of cases in only 22 weeks reported.

Through the Report of the Multi‐year National Plan of the Public Sector 2017–2021, the MEPyD of the government of the DR presented the results of the measures aimed at ensuring DR agriculture is competitive and sustainable. The report, however, did not provide descriptive results of initiatives aimed at public health, food safety, and traceability. Similarly, while training for rural populations (<1200 people) is mentioned, the topics addressed in the training are not specified, so it is difficult to determine whether and how many producers have received training in key food safety areas such as good agricultural practices (GAPs) and good manufacturing practices (GMPs). For its part, the manufacturing sector reported a reduction (−4%) in 2019 in access to GMPs training, a decrease attributed to the lack of personnel needed to coordinate and execute training (Ministerio de Economía Planificación y Desarrollo [MEPyD], 2019), showing that the government's intention to create an agri‐food health and safety system that includes surveillance and training of all representatives of the production chain is a necessity that requires concrete actions to reduce contamination of food.

The Ministry of Agriculture in 2020 reported the training of 3122 producers and technicians in GAPs at the national level during the 2016–2020 period and the implementation of a national phytosanitary surveillance system, and other projects focused on food quality and safety of some specific products such as banana, pineapple, and cassava. The ministry issued 26,586 phytosanitary certificates for export agricultural products and carried out 131 risk analyses for products imported into the DR in 2020. In relation to the animal health program, it also reported 1,973,997 pigs vaccinated against classical swine fever, 14,010 bovine farms with known status for bovine brucellosis and tuberculosis during the 2016–2020 period (Ministerio de Agricultura, 2020). However, no integrated, modern, and efficient control and surveillance program was reported, with a dynamic approach to training, monitoring and support, involving all sectors of the production chain in search of preserving the health of consumers.

In addition to the official reports of the government of the DR in relation to the efforts and investigations on food safety, the authors are unaware of the existence of current scientific works, between 2012 and 2022, in which farmers, distributors, or consumers are the focus of research to determine the level of knowledge about food safety, use of GAPs, food safety practices, Hazard Analysis and Critical Control Points or other systematic approaches to ensuring food safety.

### 3.11 Expectations and challenges—food security in the Dominican Republic

Food safety efforts in the DR should focus on integrated, modern, and efficient plans and projects that operate from different fronts of the national and international order to combat the multiple obstacles to improve food safety. The DR already has laws and a legislative structure and, in some cases, enforcement capacity aimed at improving food safety. Robust methods to monitor and enforce noncompliance with established standards, however, need to be strengthened. This requires a government commitment and public spending that must be seen as an investment for social welfare that will result in decreases in public spending on medical care for foodborne diseases, diseases associated with poor diet, decreases in expenses for school repetition, as well as greater productivity due to the reduction in absenteeism (Prost & Martínez, 2019).

Efforts to improve rural infrastructure and support services for food production should be strongly considered. The promotion of GAPs to eliminate contamination at the earliest stages of food production; promotion of innovation and adoption of food safety technologies; and improved public‐private alliances in the agricultural sector for the commercialization of agricultural products in safe conditions, would each bolster food safety across the country.

To guide training programs for producers on GAPs in the DR, it is necessary to promote research able to precisely identify the type of pathogens responsible for diseases transmitted by food and water in the DR and how those pathogens enter food supplies. Doing so would identify the critical control points and allow for risk‐based practices that more effectively utilize resources.

It is also necessary to promote research that identifies barriers to the adoption of food safety practices. At the same time, research that defines the conditions necessary to facilitate adoption of food safety practices could create an enabling environment for food safety improvements.

In this context, the universities and educational institutions in the DR should contribute to food safety improvements, since both the generation of knowledge on food safety and the use of scientific data that facilitate the adoption of new behaviors that result in food production under food safety standards require the training of food scientists who are very aware of local contexts and trained to carry out research on food safety within the context of the DR. Food safety research must consider that both the production chains, the different forms of food marketing and distribution, and even the forms of food consumption in the DR are specific and, in this situation, determine measures that are truly applicable and durable over time to improve food safety in the DR.

The Ministry of Agriculture of the DR government has developed some projects related to food health and safety since 2011, such as the Agrifood Competitive Transition Support Program (PATCA) for a value of 11 million dollars and the project “Improvement of Agrifood Health and Safety in the Dom. Rep.(PATCA III)” of 2019 with a total cost of 50 million dollars with an execution date until December 2024 (Ministerio de Agricultura, n.d.). It would be of great benefit to link these projects to national universities to achieve the training of professionals who will then be in charge of generating knowledge and providing education and training to stakeholders.

Additionally, it is necessary to strengthen control and surveillance mechanisms that are aligned with the conditions of production, distribution, and marketing of food products in the DR. Available models are often developed for countries with vastly different food production systems and consumer behaviors, and, thus, different food safety challenges. Likewise, many existing models were created for countries with greater resources to implement such programs making the sustainability of borrowed models a challenge for countries with fewer support resources. Thus, these surveillance and monitoring programs should be crafted to be effective within the DR context as well.

Finally, countries with healthy and collaborative relationships between the public sector, private sectors, and universities (Etzkowitz, 2008) are often best‐positioned to address national challenges such as food safety. In this aspect, the DR could consider bolstering food safety capacity (teaching, research, engagement) at the university level. Currently, there is very little active research on food safety as it relates to the Dominican Republic context (e.g., food produced/consumed in the DR using DR production practices, etc.). A search of the Web of Science database using the terms “food safety” + “Dominican Republic” produced only 16 publications (6800 records were retrieved for the same period using “food safety” + “United States”). Likewise, published research on food safety in the DR is very often conducted and authored by non‐Dominicans. While such research is beneficial, the number of studies is still quite low and the lack of Dominican‐led food safety research programs and the integration of these results into policy development is a barrier to improving food safety in the DR.

## 4 CONCLUSION

Food security in the DR has been a constant concern of the national government and international organizations that have worked to create more favorable conditions for the country's population. However, efforts in access and availability are offset by the number of cases of food and waterborne illnesses and the almost nonexistent information provided by the government on the pathogens responsible for these cases.

This review presents an updated overview of the state of food safety in the DR, including the current state of laws, regulations, and control agencies responsible for supervising and monitoring food safety, the most recent factors that impact food safety such as tourism, the COVID‐19 pandemic, food alternatives associated with safety and the perception of safety.

The review also considers the influence of food availability and sufficiency in the poorest populations of the country, since this directly affects the perception and importance of food safety in the population, making it clear that in most cases, limits to accessibility outweigh food safety concerns. Thus, improving food safety or nutritional quality requires plans to reduce inequality in the population and to be able to guarantee food security for all. Finally, the DR must implement integrated, modern, and efficient systems and programs able to combat the multiple financial, structural, and behavioral obstacles that can exacerbate food safety challenges.

## AUTHOR CONTRIBUTIONS

**Silvia J. R. Vargas:** Data curation (equal); formal analysis (equal); investigation (equal); writing – original draft (equal); writing – review and editing (equal). **Patricia Sipes:** Investigation (equal); writing – original draft (equal); writing – review and editing (equal). **Silvia Tortosa la Osa:** Investigation (equal); writing – original draft (equal); writing – review and editing (equal). **Paul Ebner:** Conceptualization (equal); data curation (equal); formal analysis (equal); investigation (equal); methodology (equal); writing – original draft (equal); writing – review and editing (equal).

## FUNDING INFORMATION

This study was made possible by the generous support of the United States Department of Agriculture through Cooperative Agreement No. OGSM: FCC‐517‐2020/001‐00 to Improving Economies for Stronger Communities (IESC) and subaward to Purdue University. The contents are the responsibility of the authors and do not necessarily reflect the views of IESC or the United States Government.

## CONFLICT OF INTEREST STATEMENT

The authors declare that they have no known competing financial interests or personal relationships that could have appeared to influence the work reported in this paper.

## Supporting information


Table S1. and S2.

## Data Availability

No large quantitative datasets were generated from this research.
