# Supplementary material for: Food safety in the Dominican Republic—The current situation and challenges in the public management system
Source: Food Sci Nutr. 2024 Sep 24;12(11):8608–22. doi: 10.1002/fsn3.4311 (PMC11606802; doi:10.1002/fsn3.4311)
Supplement: Supplementary file 1 — Table S1. and S2. [file FSN3-12-8608-s001.docx]

Supplemental Information

Food Safety in the Dominican Republic - The Current Situation and Challenges in the Public Management System

Vargas, Silvia J. R^1^ rodrigsj@purdue.edu

Sipes, Patricia ^2^ tsipes@purdue.edu

Tortosa la Osa, Silvia ^3^ slaosa@iesc.org

Ebner, Paul^1*^ pebner@purdue.edu

^1^ Department of Animal Sciences, Purdue University, West Lafayette, IN, 47907, USA

^2^ International Programs in Agriculture, Purdue University, West Lafayette, IN 47907, USA

^3^ Improving Economies for Stronger Communities, Trade Safe (TraSa) Project, Santo Domingo, Dominican Republic

*** Correspondence:**Corresponding Author
pebner@purdue.edu

| *Table S1. Cases of diarrheal diseases associated with food and water consumption in Dominican Republic between 2012 - 2018 ** | | | | | | | |
| --- | --- | --- | --- | --- | --- | --- | --- |
| Food and waterborne illnesses | 2012 | 2013 | 2014 | 2015 | 2016 | 2017 | 2018 |
| Acute diarrheal disease | 600910 | 569072 | - | 832304^a^ | 832099^b^ | 575747 | 774967 |
| Foodborne illness | 20793 | 23925 | - | - | - | 23648 | 23570 |
| Cholera | 458 | 158 | 558 | 544 | 1159 | 122 | 118 |
| Leptospirosis | 217 | 112 | 538 | 466 | 779 | 792 | 580 |
| - No data | | | | |  |  |  |
| ^a^- Value reported only in the weekly report N°52 | | | | |  |  |  |
| ^b^ - Sum of cases in only 22 weeks reported | | | | |  |  |  |
| *Data extracted from the epidemiological reports weeks of the Ministry of Public Health of the Dominican Republic, consulted 4/25/2022 <https://digepi.gob.do/documentos/>   \| *Table S2. Annual cases of acute diarrheal disease and foodborne illness x 100,000 inhabitants in Dominican Republic 2012-2018.* \| \| \| \| \| \| \| --- \| --- \| --- \| --- \| --- \| --- \| \| Year \| Population DR* \| ADD episodes \| ADD annual cases x 100000 inhabitants \| Foodborne illness cases \| Foodborne illness cases x 100000 inhabitants \| \| 2012 \| 9680963 \| 600910 \| 6207 \| 20793 \| 215 \| \| 2013 \| 9883486 \| 569072 \| 5758 \| 23925 \| 242 \| \| 2014 \| 9883486 \| - \| - \| - \| - \| \| 2015 \| 9980243 \| 832304 \| 8340 \| - \| - \| \| 2016 \| 10075045 \| 832099 \| 8259 \| - \| - \| \| 2017 \| 10169172 \| 575747 \| 5662 \| 23648 \| 233 \| \| 2018 \| 10266149 \| 774967 \| 7549 \| 23570 \| 230 \| \| *National Statistical Office in DR (ONE); ADD = acute diarrheal disease; “-” = no data \| \| \| \| \| \| \|  \| \|  \|  \|  \|  \| | | | | | | | |
